# Therapeutic Effects of Human Urine-Derived Stem Cells in a Rat Model of Cisplatin-Induced Acute Kidney Injury In Vivo and In Vitro

**DOI:** 10.1155/2019/8035076

**Published:** 2019-11-22

**Authors:** Bishao Sun, Xing Luo, Chengfei Yang, Peilin Liu, Yang Yang, Xingyou Dong, Zhenxing Yang, Jie Xu, Yuanyuan Zhang, Longkun Li

**Affiliations:** ^1^Department of Urology, Second Affiliated Hospital, Army Medical University, Chongqing 400037, China; ^2^Wake Forest Institute for Regenerative Medicine, Wake Forest School of Medicine, Winston-Salem, North Carolina 27157, USA

## Abstract

Acute kidney injury (AKI) is an extremely dangerous clinical syndrome with high morbidity and mortality. Stem cell-based therapies have shown great promise for AKI treatment. Urine-derived stem cells (USCs) are a novel cell source in tissue engineering and cell therapy which provide advantages of simple, noninvasive, and low-cost harvest methods, efficient proliferation, and multi-differentiation potential. Here, we described the therapeutic effects of USCs in a rat model of cisplatin-induced AKI as a novel therapy. In vivo, the intravenous administration of USCs alleviated the renal functional damage in AKI rats, for the levels of blood urea nitrogen (BUN) and serum creatinine (SCr) were significantly decreased. The USCs-treated group also exhibited improved histological and ultrastructural changes, promoted proliferation, and inhibited apoptosis in renal tissues. After the USC therapy, the expression levels of proinflammatory cytokines (TNF-*α* and IL-6) and apoptosis-related proteins (BAX and cleaved caspase-3) were downregulated. In addition, the presence of a few GFP-labeled USCs was confirmed in rat renal tissues. In vitro, rat tubular epithelial (NRK-52E) cells were incubated with cisplatin to induce cell damage and then cocultured with USCs. After coculture with USCs, the cisplatin-induced NRK-52E cells showed higher cell viability and a lower apoptosis ratio than those of the control group, and cell cycle arrest was improved. In conclusion, our results demonstrated that USC therapy significantly improved the renal function and histological damage, inhibited the inflammation and apoptosis processes in the kidney, and promoted tubular epithelial proliferation. Our study exhibited the potential of USCs in the treatment of AKI, representing a new clinical therapeutic strategy.

## 1. Introduction

Acute kidney injury (AKI) is a growing health concern worldwide because of its dramatic rise in incidence and its adverse outcomes [1]. The primary risk factors for AKI include diabetes, hypertension, and advanced age, which huge numbers of people are suffering from or facing [2]. Rapid increases in the incidence of AKI have been reported recently, and the condition is more severe among hospitalized patients [3]. AKI affects up to 50% of critically ill patients and increases short- and long-term mortality risk; the most frequent causes of AKI in the critically ill are sepsis, hypovolemia, and nephrotoxic agents [4]. Therefore, animal models simulating these processes (including ischemia/reperfusion injury and nephrotoxic agent administration) are widely used for pathological and therapeutic studies. Despite recent advances in medicine, few interventions can be used to treat AKI except supportive therapies such as dialysis and renal replacement therapy. More resources are needed to be directed toward AKI treatment and prevention.

Cisplatin is a first-line anticancer drug widely used for the treatment of various solid tumors, but it is also nephrotoxic [5]. Cisplatin acts via interacting with DNA, thus leading to DNA damage and mitochondrial dysfunction; it tends to accumulate in renal tubular cells during drug metabolism, thus causing cell death and resulting in AKI [5]. Renal tubular damage is the key pathological alteration of AKI, and the regeneration of impaired tubular epithelial cells is crucial for recovery [6]. Novel stem cell-based therapies, such as the administration of bone marrow-derived mesenchymal stem cells (BMSCs), umbilical cord blood-derived mesenchymal stem cells (UC-MSCs), adipose-derived mesenchymal stromal/stem cells (AMSCs), and induced pluripotent stem cells (iPSCs), have shown great promise for the treatment of AKI [7–11]. However, there are disadvantages to the clinical use of these stem cells, e.g., the isolation and culture of mesenchymal stem cells (MSCs) is usually invasive, slow, and expensive, and the application of MSCs for allotransplantation raises immunological rejection and ethical issues [12], and iPSCs can be generated from one's somatic cells, but they remain potentially tumorigenic. More useful stem cells are required for basic studies and clinical applications.

Urine-derived stem cells (USCs) are novel adult stem cells that are isolated from urine and are harvested through a method that is noninvasive, efficient, and affordable [13]. USCs can be obtained easily regardless of the donor's age, gender, or health condition (except for some special conditions, including urinary tract infections and anuria) and are able to differentiate into common cell types of the urinary tract, such as smooth muscle cells (SMCs), urothelial cells (UCs), and endothelial cells, with high efficiency [14]. In addition, USCs originate from the kidney and are considered to be an ideal cell source for therapy for urinary tract diseases [15–17]. Interestingly, it was reported that USCs contributed to the repair of ischemia/reperfusion renal injury in rat models which represent the prerenal (i.e., hypoperfusion) AKI [18]. Moreover, the exosomes secreted by USCs were confirmed to be useful in the prevention of renal complications from type I diabetes in rats [19]. USCs are shown to be a good therapy in different kinds of renal injuries, but little is known about their effects in intrarenal (i.e., nephrotoxic agents) AKI models; the present study was designed to explore the potential therapeutic effects of USCs on rats with cisplatin-induced AKI in vivo and in vitro.

## 2. Materials and Methods

### 2.1. Ethics Statement

The research was conducted in accordance with the ethical standards detailed in the Declaration of Helsinki, and it was approved by the Medical Ethics Committee of the Second Affiliated Hospital (Xinqiao Hospital) of Army Medical University (Chongqing, China). Written informed consent was obtained from every donor prior to donating urine samples.

### 2.2. Isolation and Culture of USCs

USCs were isolated from the urine of 6 healthy adult donors with an average age of 22.67 ± 1.49 years old according to the methods described in previous studies [20, 21]. Briefly, sterile midstream urine samples were collected and centrifuged, and the cell pellets were washed and resuspended for further culture. Primary medium was used for the first three days, and RE/MC medium was used for further proliferation culture. The primary medium was made up of a 1 : 1 mix of high-glucose DMEM (HyClone, Utah, USA) and Ham's F12 nutrient (Gibco, Grand Island, USA) supplemented with 10% FBS (Gibco, Australia), 1% antibiotic-antimycotic (Gibco, Grand Island, USA), and the components of the REGM SingleQuot kit (Lonza, Basel, Switzerland). The RE proliferation medium contained 500 ml of RE cell basal medium supplemented with the components of the REGM SingleQuot kit. The MC proliferation medium contained high-glucose DMEM supplemented with 10% FBS, 1% GlutaMAX (Gibco, Japan), 1% NEAA (Gibco, Grand Island, USA), 1% pen/strep (Gibco, Grand Island, USA), 5 ng/ml bFGF (PeproTech, Rocky Hill, USA), 5 ng/ml PDGF-AB (PeproTech, Rocky Hill, USA), and 5 ng/ml EGF (PeproTech, Rocky Hill, USA). The RE/MC medium was made up of a 1 : 1 mix of RE proliferation medium and MC proliferation medium. Cells from the P3 generation were used for downstream experiments.

### 2.3. Cell Proliferation Assay

Cell proliferation was assessed using the Cell Counting Kit-8 (CCK-8) reagent (MCE, Shanghai, China) according to the manufacturer's instructions. Cells were seeded in 96-well plates at a density of 2000 cells per well. CCK-8 solution (10 *μ*l/well) was added to the medium, after which the cultures were incubated for an additional 2 h at 37°C. The absorbance was measured at a wavelength of 450 nm using a microplate reader (Thermo Fisher, USA). All experiments were performed in triplicate and were repeated three times, and then the growth curve was generated.

### 2.4. Flow Cytometry Assay and Antibodies

For surface marker detection, USCs were collected and resuspended at a density of 1 × 10^8^ cells/ml, and then 100 *μ*l of the resuspended cells was aliquoted into tubes. The following monoclonal antibodies were used: anti-human CD31 eFluor 710 (eBioscience, 46-0319-42), anti-human CD34 PE (eBioscience, 12-0349), anti-human/mouse CD44 PE (eBioscience, 12-0441), anti-human CD73 FITC (eBioscience, 11-0739), anti-human CD105 APC (eBioscience, 17-1057-42), and anti-human CD146 FITC (eBioscience, 11-1469). After incubation for 30 min at 37°C, the cells were washed twice with phosphate-buffered saline (PBS) and then resuspended in 300 *μ*l of PBS for flow cytometry. For the cell cycle test, more than 1 × 10^6^ cells were collected, 1 ml of precooled 70% alcohol was added for overnight fixation, and the distribution of the cell cycle was detected the next day. For apoptosis detection, more than 1 × 10^6^ cells were collected, and the Annexin V-FITC/PI kit (Beyotime, Shanghai, China) was used according to the manufacturer's protocol.

### 2.5. Cell Immunofluorescence (IF) Staining and Antibodies

USCs were assessed for the expression of the stem cell surface markers CD31 (Cell Signaling Technology, 3528), CD34 (Proteintech, 14486-1-AP), CD45 (Cell Signaling Technology, 13917), CD44 (Abcam, ab46793), CD133 (Proteintech, 18470-1-AP), SSEA4 (Proteintech, 19497-1-AP), CD146 (Proteintech, 17564-1-AP), platelet-derived growth factor beta-receptor (PDGFRB, Proteintech, 13449-1-AP), and neural/glial antigen 2 (NG2, Abcam, ab129051), and the SMC- or UC-induced USCs were evaluated for the SMC or UC surface marker desmin (Abcam, ab32362), myosin (Proteintech, 20716-1-AP), alpha-smooth muscle actin (ASMA, Proteintech, 23660-1-AP), vimentin (Proteintech, 10366-1-AP), or uroplakin 1A (UP-Ia, Proteintech, 25275-1-AP), uroplakin 3 (UP-III, Proteintech, 15709-1-AP), cytokeratin clone AE1/AE3 (AE1/AE3, Proteintech, 18566-1-AP), and cytokeratin 13 (CK13, Proteintech, 10164-2-AP). Briefly, the slides were fixed with 4% paraformaldehyde (Boster, Wuhan, China) for 20 min at room temperature (RT) and washed with PBS three times. The cells were permeabilized with 0.1% Triton-X 100 in PBS for 10 min and blocked with 3% donkey serum (Gibco, Grand Island, USA) in PBS for 30 min. The slides were incubated with the abovementioned primary antibodies overnight at 4°C. After washing three times, the slides were incubated with the appropriate secondary antibody (Thermo Fisher; A-11001, A-11034, A-21235, and A-21244) at RT for 45 min in the dark. The slides were mounted using antifade mounting media (Invitrogen, USA), and images were captured using a laser-scanning confocal microscope (Leica, SP5, Germany).

### 2.6. SMC and UC Differentiation

USCs plated at a density of 1000 cells/cm^2^ were used for differentiation into two lineages by culturing in a specific medium for 14 days. For myogenic differentiation, equal volumes of DMEM containing 10% FBS and embryonic fibroblast medium (Cyagen, Guangzhou, China) containing 10% FBS, 2.5 ng/ml TGF-*β*1 (PeproTech, Rocky Hill, USA), and 5 ng/ml PDGF-BB (PeproTech, Rocky Hill, USA) were used. For uroepithelial differentiation, DMEM containing 10% FBS was mixed with KSFM (Gibco, Grand Island, USA) at a 1 : 4 ratio, and 30 ng/ml EGF was added to the mixture. The differentiation medium was replaced every 3 days.

### 2.7. Green Fluorescent Protein (GFP) Transfection and Detection

USCs were transfected with a lentiviral-GFP vector (Genechem, Shanghai, China) according to the manufacturer's protocol. Briefly, USCs at a confluence 30-50% were cultured in an incubator (5% CO_2_, 37°C), the GFP lentivirus (1 × 10^8^ TU/ml) was suspended in the RE/MC medium, the RE/MC medium was replaced with the medium containing the lentivirus, and the cells were cultured for 4 h. Then, the medium was discarded, and the cells were further cultured for 48 h. Obvious green fluorescence was observed under a fluorescence microscope. The GFP-labeled USCs were injected into the model rats, and the kidney tissues were sectioned into 5 *μ*m thick samples using a cryostat microtome. After fixation in 4% paraformaldehyde for 15 min, the sections were incubated with 4′,6-diamidino-2-phenylindole (DAPI, Solarbio, Beijing, China) to stain the cell nuclei. Images were captured using a Leica laser-scanning confocal microscope.

### 2.8. Animal Models

In total, 30 Sprague-Dawley rats were randomly divided into 3 groups: the normal control group (normal, *n* = 10), the AKI group treated with PBS (AKI+PBS, *n* = 10), and the AKI group treated with USCs (AKI+USCs, *n* = 10). Cisplatin (MCE, Shanghai, China) was dissolved in normal saline (1 mg/ml), and the AKI model was induced by the intraperitoneal injection of cisplatin at a dosage of 5 mg/kg body weight, while the normal control rats received a saline injection only. The date of model establishment date was considered d0. The AKI+USCs group rats received an injection of USCs (2 × 10^6^ cells suspended in 0.2 ml of PBS), while the AKI+PBS group received an injection of 0.2 ml of PBS the next day (d1). The rats were anesthetized with isoflurane through a breathing mask (RWD Life Science, Shenzhen, China), and the cell suspension was injected into the tail vein. GFP-labeled USCs were detected at d2 and d4. All rats were sacrificed at d4. Blood was sampled from the eyes of the rats, and blood urea nitrogen (BUN) and serum creatinine (SCr) were detected. The left kidney was fixed in a paraformaldehyde or glutaraldehyde fixing solution for morphological staining and transmission electron microscopy (TEM), and the right kidney was used for the extraction of total protein.

### 2.9. Histological Staining and Morphological Evaluation

The kidney tissues were embedded in paraffin after fixation and sectioned into 5 *μ*m thick sections. The sections were stained with hematoxylin and eosin (HE) and periodic acid-Schiff (PAS) reagents, and all sections were evaluated under a light microscope by a pathologist who was blinded to the treatment groups. The degree of renal tubular injury was scored by reported methods [18]. 10 high-magnification visual fields (×200) of HE staining were randomly selected from the renal medulla of each rat, and 10 renal tubules were selected from each visual field, a total of 100 renal tubules were scored: obvious dilation of renal tubules, flattened cells, or swelling (1 point); brush edge damage or fall off (1~2 points); appearance of tube type (2 points); and lumen with exfoliated necrotic cells (no tubular shape or debris) (1 point). The glomerular injury was semiquantified by counting. 3 high-magnification visual fields (×200) in the cortex area were randomly selected from each rat, and the injured glomeruli were counted in each visual field, then the average number was calculated. Glomeruli containing any of the following lesions were included in the calculation: glomerular atrophy, congested glomerular capillary plexus, endothelial edema and vacuolization, mesangial cell proliferation and swelling, and matrix expansion. The PAS-positive area ratio was calculated using Image-Pro Plus 6.0 software (Media Cybernetics Inc., Rockville, MD, USA). A very high-magnification visual field (×400) was used, and 5 glomeruli were randomly selected. The positive area of glomeruli along with the whole area of glomeruli was measured, and the ratio of PAS-positive area to glomerular area was calculated. At least 3 rats of each group were used for the above morphological evaluations.

### 2.10. TEM

After the rats were sacrificed, renal tissue samples were harvested immediately. Small pieces (approximately 1 mm^3^) of renal cortex tissue were fixed in a glutaraldehyde fixing solution for 24 h at 4°C. The tissues were then washed and postfixed for 2 h in 1% osmium tetroxide at 4°C. The specimens were washed again and dehydrated with acetone, then were soaked in an embedding medium. Sections at a thickness of 60~90 nm were cut and then stained with uranyl acetate and lead acetate. The ultrastructure of the kidney was observed under a TEM machine (Hitachi Ltd., Tokyo, Japan).

### 2.11. Immunohistochemistry (IHC) Staining

To evaluate the proliferation of the kidney, the expression of Ki67 was assessed by immunohistochemistry. The PFA-fixed, paraffin-embedded sections were deparaffinized and rehydrated first, and heat-mediated antigen retrieval was performed using Tris/EDTA buffer, pH 9.0. Then, the sections were treated with 3% H_2_O_2_ for 30 min and incubated with an ImmunoBlock reagent for 30 min at RT. The sections were incubated with a Ki67 primary antibody (Abcam, ab92742) at 4°C overnight, followed by biotinylated anti-rabbit IgG secondary antibody for 30 min at 37°C. The antigen-antibody reactions were visualized with diaminobenzidine (DAB), which resulted in a brown-colored precipitate at the antigen site, and hematoxylin counterstaining was performed. Twenty random fields from each section were photographed, and semiquantitative evaluations were performed using ImageJ software (Rawak Software Inc., Stuttgart, Germany).

### 2.12. TUNEL Assay

DNA cleavage was detected using fluorescein TUNEL assays (Roche Diagnostics, Mannheim, Germany) according to the manufacturer's protocol. Briefly, sections were deparaffinized and rehydrated, and then a proteinase K working solution was used for antigen retrieval. The TUNEL reaction was conducted by mixing the reagents of the kit after permeabilization. After DAPI counterstaining of the nuclei, the stained sections were examined by microscopy, and the number of apoptotic cells in 20 random fields was calculated under a magnification of 60×.

### 2.13. Western Blot Analysis and Antibodies

Total protein was extracted from the kidney tissues using the RIPA lysis buffer (Beyotime, Shanghai, China), and the concentration was measured with a BCA Assay Kit (Beyotime, Shanghai, China). Then, 30 *μ*g of protein was separated using 8-12% sodium dodecyl sulfate polyacrylamide gel electrophoresis (SDS-PAGE) and transferred onto polyvinylidene fluoride (PVDF) membranes (Merck Millipore, Darmstadt, Germany). After being blocked with 5% skim milk (dissolved in Tris-buffered saline) at RT for 2 h, the membranes were incubated overnight at 4°C with the following primary antibodies: IL-6 (Proteintech, 21865-1-AP), TNF-*α* (Proteintech, 17590-1-AP), NF-*κ*B (Cell Signaling Technology, 6956), caspase-3 (Proteintech, 19677-1-AP), BCL-2 (Proteintech, 12789-1-AP), BAX (Proteintech, 50599-2-Ig), GAPDH (Proteintech, 60004-1-Ig), and *β*-actin (Beyotime, Shanghai, China, AF0003). Then, the membranes were hybridized with horseradish peroxidase-conjugated secondary antibodies (Zhongshan Co., Beijing, China, ZB-2301, and ZB-2305). An ECL substrate (Millipore, Billerica, USA) was used to detect the protein bands, and the images were visualized with an ImageQuant LAS-4000 BioImaging System (GE Healthcare, Stockholm, Sweden).

### 2.14. The Induction of NRK-52E Cells with Cisplatin

The rat kidney epithelial cell line NRK-52E was purchased from Zhong Qiao Xin Zhou Biotechnology Co., Ltd. (Shanghai, China) and cultured in high-glucose DMEM containing 10% FBS. When the cells reached 60-70% confluence, gradient concentrations of cisplatin (0 *μ*M, 2.5 *μ*M, 5 *μ*M, 10 *μ*M, 20 *μ*M, and 40 *μ*M) were applied for 16 h. The CCK-8 test and flow cytometry were conducted at the end of the induction to detect cell viability and cell cycle distribution. The concentrations that led to evident cell damage were used for subsequent experiments.

### 2.15. Coculture of NRK-52E Cells and USCs

The NRK-52E cells were cultured in 6-well plates. After being induced with cisplatin for 16 h, the medium was replaced, and the NRK-52E cells were noncontact cocultured with USCs (5 × 10^5^ cells) in the logarithmic phase using inserts with a 24 mm diameter and 0.4 *μ*m pores in 6-well plates (Corning, Kennebunk, USA). Twenty-four hours after coculture, the NRK-52E cells were collected for the CCK-8 test, and apoptosis and cell cycle were detected using flow cytometry. Twenty-four-hour cultures of induced NRK-52E cells were used as a negative control.

### 2.16. Statistical Analysis

The data were presented as the mean ± standard error of the mean. Statistical analyses were performed with SPSS 19.0 software (SPSS Inc., Chicago, USA), and significant differences between each group were calculated using analysis of variance or the Mann-Whitney *U* test. *P* < 0.05 was considered statistically significant. All experiments were performed with no less than three independent replications.

## 3. Results

### 3.1. Growth Characteristics and Multipotential Differentiation of USCs

**Figure 1:**
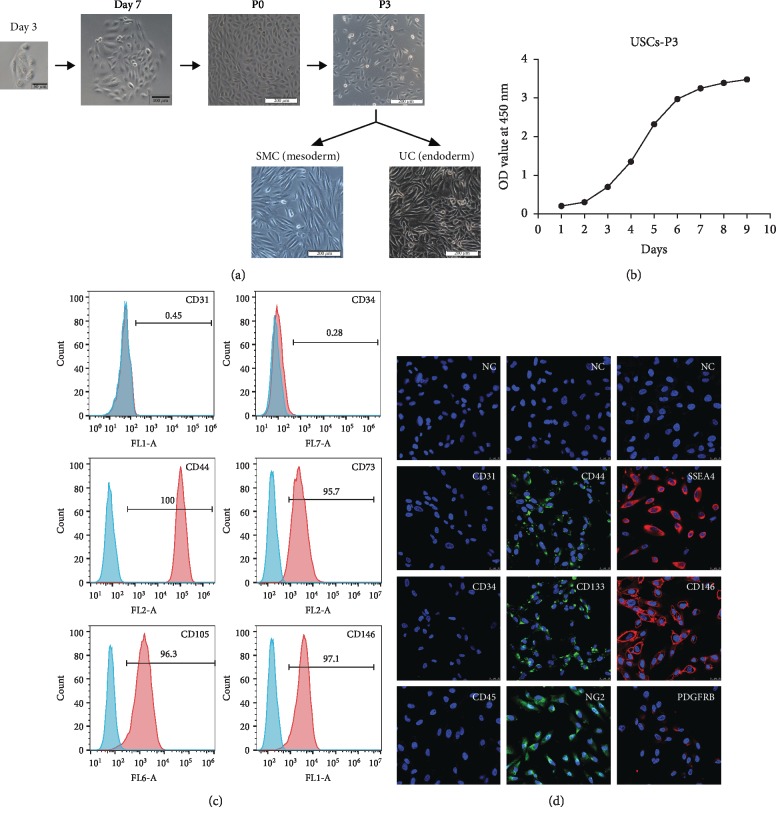
Growth characteristics of USCs. (a) The morphology of USCs by passage and differentiation. Single, small, compact rice grain-like cells were observed on the third day after initial seeding, and they formed a colony on the seventh day. The cells were considered to be at P0 when the confluence reached 70-80% and were passaged to the next generation. The USCs maintained the rice grain-like morphology after several passages, and USCs from the P3 generation were induced to differentiate into SMCs and UCs. The cells showed an elongated and spindle-shaped morphology after SMC differentiation and a cobblestone-shaped morphology after UC differentiation. Scale bar: 50 *μ*m, 100 *μ*m, and 200 *μ*m. (b) The growth curve of USCs from the P3 generation. (c) Detection of surface markers in USCs using flow cytometry. USCs did not express hematopoietic stem cell markers (CD31: 0.45%, CD34: 0.28%) but expressed MSC markers (CD44: 100%, CD73: 97.1%, and CD105: 96.3%) and pericyte markers (CD146: 95.7%). (d) Detection of surface markers in USCs using IF. USCs did not express hematopoietic stem cell markers (CD31, CD34, and CD45) but did express MSC markers (CD44 and CD133), the ESC marker SSEA4, and pericyte markers (CD146, PDGFRB, and NG2). NC: negative control; PDGFRB: platelet-derived growth factor beta-receptor; NG2: neural/glial antigen 2. Scale bar: 25 *μ*m.

**Figure 2:**
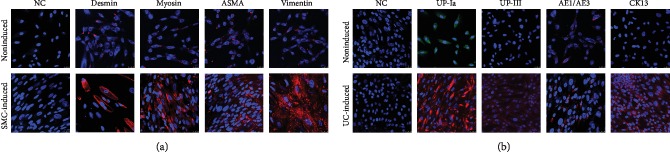
Multipotential differentiation of USCs. (a) SMC-differentiated cells showed an increased expression of the SMC-specific markers desmin, myosin, ASMA, and vimentin. (b) The UC-differentiated cells showed an increased expression of the UC-specific markers UP-Ia, UP-III, AE1/AE3, and CK13. NC: negative control; ASMA: alpha-smooth muscle actin; UP-Ia: uroplakin 1A; UP-III: uroplakin 3; AE1/AE3: cytokeratin, clone AE1/AE3; CK13: cytokeratin 13. Scale bar: 25 *μ*m.

A small cluster of rice grain-like cells was observed 2-3 days after initial seeding, and rapidly formed clones were observed within an additional 4-6 days. The cells maintained the rice grain-like morphology after several passages (Figure 1(a)). The CCK-8 test confirmed logarithmic growth in the USCs (Figure 1(b)). Flow cytometry and IF confirmed that MSC-like cell surface markers (CD44, CD73, CD105, and CD133) but not hematopoietic stem cell markers (CD31, CD34, and CD45) were consistently expressed in the USCs. Additionally, USCs expressed the embryonic stem cell (ESC) marker SSEA4 and pericyte markers (CD146, PDGFRB, and NG2) (Figures 1(c) and 1(d)). More importantly, USCs had the ability to differentiate into a smooth muscle lineage (mesodermal origin) and urothelial lineage (endodermal origin) after induction for 14 days. The induced cells showed an SMC-like (elongated and spindle-shaped) or UC-like (cobblestone-shaped) morphology (Figure 1(a)) and increasingly expressed SMC surface markers (desmin, myosin, ASMA, and vimentin) (Figure 2(a)) or UC-specific surface markers (UP-Ia, UP-III, AE1/AE3, and CK13) (Figure 2(b)), respectively. The USCs we obtained showed MSC-like features and the potential for urological application, which is consistent with our previous reports [13].

### 3.2. The Effects of USCs on Renal Function and Histological Damage

**Figure 3:**
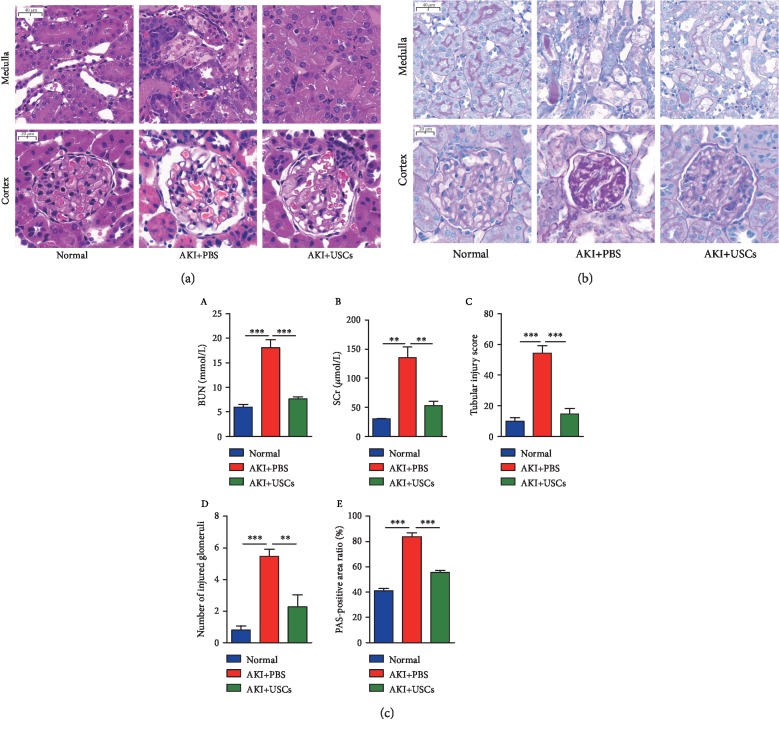
USCs improved renal function and histological damage in AKI rats. (a) HE staining of kidney tissues. (b) PAS staining of kidney tissues. (c) Statistical analysis of renal function indicators. (A) Blood urea nitrogen (BUN). (B). Serum creatinine (SCr). (C) Tubular injury score. (D) Number of injured glomeruli. (E). PAS-positive area ratio. ^∗∗^*P* < 0.01, ^∗∗∗^*P* < 0.001. Scale bar: medulla: 40 *μ*m, cortex: 20 *μ*m.

To evaluate the recovery of renal function, the levels of BUN and SCr in the serum were examined. Both levels were significantly higher in the AKI+PBS group than in the normal group, and these biochemical values both decreased after the administration of USCs (Figure 3(c) A, B). To determine the effects of USCs on histological damage, HE and PAS staining were performed to analyze the histological alteration of kidney tissues in tissue cross-sections. In the medulla, there was a large amount of tubular necrosis with lumen expansion, brush border loss, and a large number of flattened tubular epithelial cells and casts in the AKI groups compared with the normal group, and the impairments were obviously alleviated in the AKI+USCs group compared with the AKI+PBS group (Figures 3(a) and 3(b), medulla). The tubular injury score in the AKI+USCs group was significantly lower than that in the AKI+PBS group (Figure 3(c) C). In the cortex, glomerular injury including glomerular atrophy, congested glomerular capillary plexus, endothelial edema and vacuolization, mesangial cell proliferation and swelling, and matrix expansion were observed in the AKI groups compared with the normal group, and the impairments were obviously alleviated in the AKI+USCs group compared with the AKI+PBS group (Figures 3(a) and 3(b), cortex). The number of injured glomeruli in the AKI+USCs group was significantly lower than that in the AKI+PBS group (Figure 3(c) D), and the PAS-positive area ratio was significantly reduced in the AKI+USCs group compared with the AKI+PBS group (Figure 3(c) E).

### 3.3. The Effects of USCs on Renal Ultrastructural Changes

**Figure 4:**
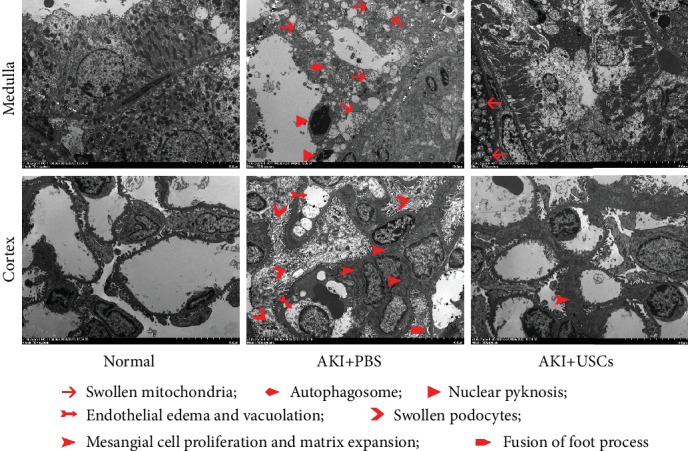
Ultrastructural changes in renal tissues. Ultrastructural changes were detected by transmission electron microscopy. Scale bar: 10 *μ*m.

TEM revealed the renal ultrastructural changes in the AKI groups, and the ultrastructural changes were improved after administration of USCs (Figure 4). In the medulla, the ultrastructure of the normal group showed intact proximal tubular epithelia with normal organelles such as abundant mitochondria, while the AKI+PBS group showed swollen mitochondria with disrupted cristae, nuclear pyknosis, and autophagosomes in tubular epithelia, and the AKI+USCs group only showed slight edema in the cytoplasm and mitochondria (Figure 4, medulla). In the cortex, the normal group showed complete three layers of filtration barrier; on the contrary, the AKI+PBS group showed endothelial edema and vacuolization, swollen podocytes, mesangial cell proliferation with matrix expansion, and segmental fusion of foot process; meanwhile, the AKI+USCs group just showed matrix expansion (Figure 4, cortex).

### 3.4. The Effects of USCs on the Proliferation and Apoptosis of the Kidney and the Tracking of GFP-Labeled USCs

**Figure 5:**
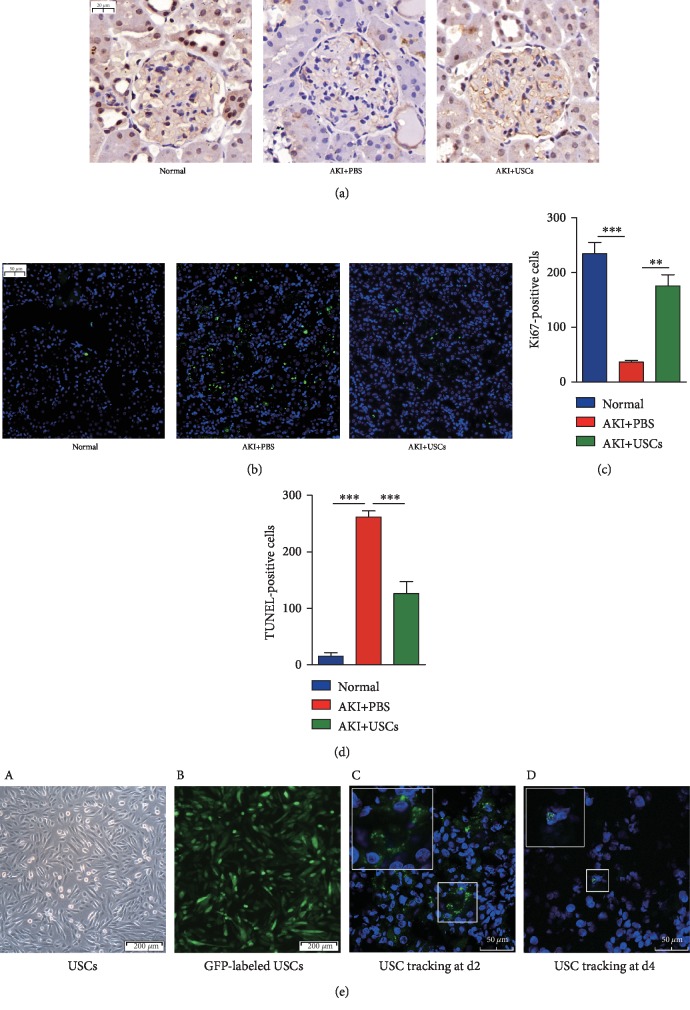
Proliferation and apoptosis of renal tissues and the tracking of USCs. (a) Proliferation was mainly observed in renal tubular epithelial cells and was increased after USC treatment. Scale bar: 20 *μ*m. (b) Apoptosis was observed in renal tissues and was decreased after USC therapy. Scale bar: 50 *μ*m. (c) Statistical analysis of Ki67-positive cells. (d) Statistical analysis of TUNEL-positive cells. (e) USCs from the P3 generation were transfected with a lentiviral-GFP vector, and the GFP protein was stably expressed in over 90% of USCs. After the tail vein injection of the GFP-labeled USCs, GFP-positive USCs were observed in the frozen sections of the kidney tissues. (A) USCs under bright field. (B) GFP-labeled USCs under a fluorescence microscope. Scale bar: 200 *μ*m. (C) A small number of GFP-labeled USCs were detected at d2. (D) Only a few GFP-labeled USCs were observed at d4. Scale bar: 50 *μ*m. GFP: green fluorescent protein; ^∗∗^*P* < 0.01, ^∗∗∗^*P* < 0.001.

The expression level of Ki67 represents the cell proliferation level; thus, IHC for Ki67 was performed to evaluate the effect of USCs on the proliferation of the kidney. Proliferation was mainly observed in tubular epithelial cells, and proliferation was at the lowest level in the AKI+PBS group but increased after the administration of USCs (Figure 5(a)). The number of proliferating cells was higher in the AKI+USCs group than in the AKI+PBS group (Figure 5(c)). Fluorescein TUNEL assays were performed to detect the apoptosis of the kidney, and cleaved DNA was stained green. Apoptosis in renal tissues was reduced after USC treatment (Figure 5(b)). The AKI+USCs group exhibited a lower number of apoptotic cells than that in the AKI+PBS group (Figure 5(d)). The injected USCs were tracked by GFP using a laser-scanning confocal microscope (Figure 5(e) A, B), and the GFP-labeled cells in the kidney tissues were observed at d2 and d4 (Figure 5(e) C, D). But only a small number of cells were found at d4.

### 3.5. Changes in the Expression of Inflammatory and Apoptosis-Associated Proteins

**Figure 6:**
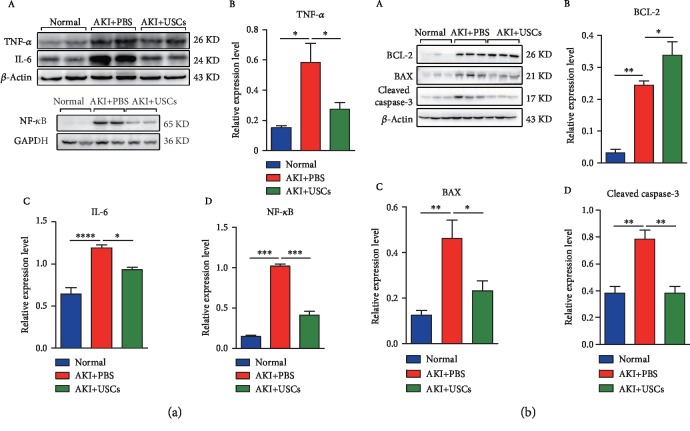
Changes in the expression of inflammatory and apoptosis-associated proteins. (a) The expression levels of inflammation-related proteins (TNF-*α*, IL-6, and NF-*κ*B) were significantly increased in the AKI+PBS group compared with the normal group and decreased in the AKI+USCs group compared with the AKI+PBS group. (A) Protein bands and statistical figures. (B) TNF-*α*. (C) IL-6. (D) NF-*κ*B. (b) The expression levels of apoptosis-related proteins (BAX and cleaved caspase-3) were significantly decreased in the AKI+USCs group compared with the AKI+PBS group, while the level of the antiapoptosis protein BCL-2 showed the reverse trend. (A) Protein bands and statistical figures. (B) BCL-2. (C) BAX. (D) Cleaved caspase-3. ^∗^*P* < 0.05, ^∗∗^*P* < 0.01, and ^∗∗∗^*P* < 0.001.

The expression levels of the inflammatory biomarkers TNF-*α*, IL-6, and NF-*κ*B in the AKI+PBS group were significantly higher than those in the normal group, and the levels in the AKI+USCs group were lower than those in the AKI+PBS group (Figure 6(a)). The expression levels of the apoptosis proteins BAX and cleaved caspase-3 were increased in the AKI+PBS group compared with those in the normal group, and the levels were significantly decreased in the AKI+USCs group compared with those in the AKI+PBS group (Figure 6(b)). However, the expression level of the antiapoptosis protein BCL-2 in the AKI+USCs group was significantly higher than that in the AKI+PBS group (Figure 6(b)).

### 3.6. The Establishment of an In Vitro Cellular Model

**Figure 7:**
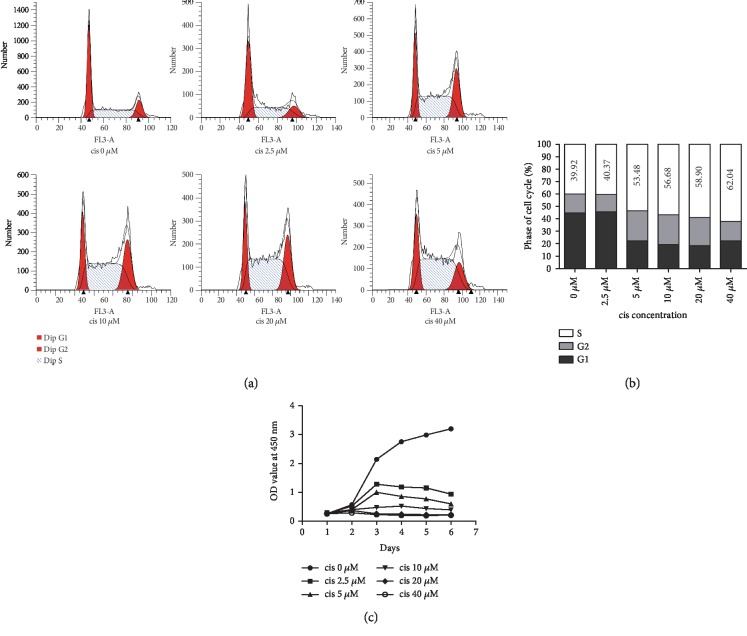
Establishment of an in vitro cellular model. (a) Cell cycle distribution. Gradient concentrations of cisplatin led to cell cycle arrest at the S phase, and the arrest became worse as the concentration increased. (b) Cell cycle distribution diagram. The numbers represent the average percentage of cells in the S phase. (c) Cell viability decreased as the concentration increased, as shown by the CCK-8 test. cis: cisplatin.

Induction with gradient concentrations of cisplatin led to different degrees of cell damage in NRK-52E cells, as shown in Figure 7. Cell cycle arrest occurred in the S phase and became worse as the concentration increased (Figures 7(a) and 7(b)). Cell viability was decreased after induction, and concentrations over 10 *μ*M led to obvious impairment (Figure 7(c)). Concentrations of 20 *μ*M and 40 *μ*M had almost equal effects (Figures 7(b) and 7(c)); thus, 10 *μ*M and 20 *μ*M were chosen as the cisplatin concentrations for in vitro induction.

### 3.7. The Effects of USCs on Induced NRK-52E Cells

**Figure 8:**
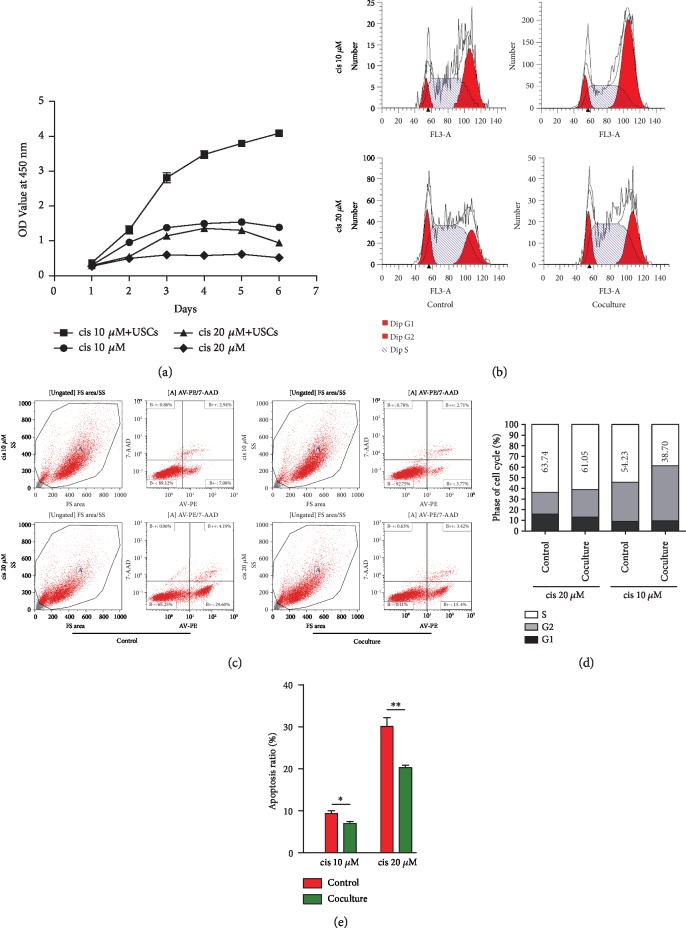
USCs improved cell viability, cell cycle arrest, and the apoptosis of cisplatin-induced NRK-52E cells. (a) Cell viability was increased after coculture with USCs, as shown by the CCK-8 test. (b) Cell cycle distribution. Cell cycle arrest at the S phase was significantly improved after coculture with USCs. (c) The apoptosis of NRK-52E cells was reduced after coculture with USCs. (d) Cell cycle distribution diagram. The numbers represent the average percentage of cells in the S phase. (e) Statistical analysis of the apoptosis ratio. cis: cisplatin; ^∗^*P* < 0.05, ^∗∗^*P* < 0.01.

NRK-52E cells were incubated with 10 *μ*M or 20 *μ*M cisplatin to induce cell damage. After coculture with USCs for 24 h, the induced cells showed higher cell viability (Figure 8(a)), and cell cycle arrest at S phase was significantly improved in the two types of induced NRK-52E cells compared with the controls (Figures 8(b) and 8(d)). In addition, the apoptosis ratio of the NRK-52E cells was remarkably decreased after coculture regardless of the preinduction concentration (Figures 8(c) and 8(e)).

## 4. Discussion

USCs are a novel type of adult stem cells and have been shown to be a remarkable cell source for cell therapy and tissue engineering, especially for urological diseases, such as diabetic cystopathy, interstitial cystitis, urethral reconstruction, and bladder reconstruction [22–25]. Although the therapeutic effects of USCs in a rat model of prerenal ischemia/reperfusion-induced AKI model have been reported, it is still necessary to investigate whether the therapeutic effects are universal in other kinds of AKI. Cisplatin-induced AKI is a typical intrarenal AKI, which is a common renal complication of cisplatin administration, and more attention should be paid to it in the clinic. Whether USCs have therapeutic effects on this type of renal injury remains unclear. In the present study, we investigated the feasibility and ability of USCs to promote the functional recovery and cellular regeneration of rat kidneys in vivo and in vitro.

Our in vivo experiments demonstrated that the intravenous administration of USCs may be an effective way to protect the rat kidney from functional and histological damage induced by cisplatin. The results revealed the anti-inflammatory, antiapoptotic, and proproliferation effects of USCs in the AKI models. Our in vitro experiments further confirmed that USCs were responsible for the proproliferation and antiapoptotic effects on renal tubular epithelial cells and that the underlying mechanism may be paracrine in nature.

In most studies on stem cell treatments, researchers have emphasized the importance of adequately targeting stem cells to injured tissues for better outcomes [26, 27]. In our experiment, most USCs were trapped in the lungs in the first 6-12 h after tail vein injection (data not shown), and this phenomenon is consistent with our previous report [22]. GFP-labeled USCs were observed in renal tissues at d2 and d4, indicating that some USCs reached the kidney and may have differentiated into renal cells and participated in repairing the injured kidney. However, only a few GFP-labeled USCs were observed at the end of the experiment, indicating that USCs may exert their effects in other ways.

An increasing number of studies have reported that exosomes may play an important role in stem cell therapy through a paracrine mechanism [28]. Exosomes are nanoscale membrane vesicles secreted from many types of cells, including stem cells, that transfer information between cells by shipping functional molecules such as DNA, RNA (mRNAs, lncRNAs, circular RNAs, and microRNAs), proteins, and lipids. These cargoes may be degraded soon when naked in the blood or other body fluids, while exosomes that consist of a phospholipid bilayer provide them a safe vehicle. Functional molecules can regulate many physiological and pathological processes, including immune response, inflammation, angiogenesis, and proliferation, in recipient cells. Researchers have reported that exosomes secreted by USCs contain various factors (such as growth factor, transforming growth factor-*β*1, angiogenin, and bone morphogenetic protein-7) and functional proteins and have therapeutic effects on various diseases, including diabetic nephropathy, diabetic skin injury, and stress urinary incontinence [19, 29, 30]. Whether exosomes secreted by USCs have the same effects on an AKI model and the pivotal role they play are worthy of further study.

The main limitation of the present study is that the relationships between therapeutic effects and the degree of injury in the model and the therapeutic regimen of stem cells are not clear, which has limited guidance for clinical application. As we know, rodents are deliberately given large doses of cisplatin in order to cause severe and easily detectable AKI. As a result, AKI appears differently in human and rat models. Various induction doses and programs could result in different degrees of injury [31]. In clinical practice, cisplatin is administered for long-term usage, at doses of less than 10 mg/kg, in patients to treat solid tumors [5]. A dose of 5 mg/kg was used in the present study, which was as close as possible to the clinical dose, but in order to avoid high death rates, cisplatin was used only once. In addition, the rat models we established were tumor-free which was quite different from clinical reality. Therefore, it is of great importance to employ tumor-bearing models with consistent administration of low dose of cisplatin in further studies. On the other hand, the therapeutic effects depend on the therapeutic regimen of stem cells. In the present study, a single intravenous injection of 2 × 10^6^ USCs generated obvious therapeutic effects. In our preliminary experiments, a lower cell number such as 5 × 10^5^ USCs could produce therapeutic effects to some extent, either. Local injection rather than intravenous injection could achieve the same goal as reported [18]. But no comparison data between the effects from different amounts or different administration routes are available. Moreover, the choice of injection time and frequency is also worth considering, e.g., when and how many injections could produce the best outcome. In general, there is still a long way to achieve the determination of the best therapeutic regimen.

Nevertheless, the present study provides an important reference for updating the treatment strategies for AKI, especially for patients with solid tumors treated with cisplatin. To avoid immune rejection, the patient's own stem cells are undoubtedly the best source of cells. Given the easily obtained nature, USCs show great application prospect in autologous transplantation [14]. USCs can be easily obtained from urine samples even in tumor patients. It has been reported that USCs from patients with bladder cancer can be successfully isolated and expanded [32]. Cisplatin is widely used for various solid tumors, such as testicular, ovarian, cervical, head, neck, non-small-cell lung, and colorectal cancers [5]. The autologous application of USCs may be used as a treatment method for AKI in these patients in the future.

## 5. Conclusion

The present study demonstrated that the administration of USCs can alleviate renal injury in AKI models in vivo and in vitro via suppressing inflammatory reactions and apoptotic processes and improving the proliferation of renal tubular epithelial cells. Further studies are needed to clarify the underlying mechanism and clinical application value.

## Data Availability

The data used to support the findings of this study are available from the corresponding authors upon request.
