# Corrigendum to “Therapeutic Effects of Human Urine-Derived Stem Cells in a Rat Model of Cisplatin-Induced Acute Kidney Injury In Vivo and In Vitro”

**DOI:** 10.1155/2021/9789181

**Published:** 2021-04-21

**Authors:** Bishao Sun, Xing Luo, Chengfei Yang, Peilin Liu, Yang Yang, Xingyou Dong, Zhenxing Yang, JieXu, Yuanyuan Zhang, Longkun Li

**Affiliations:** ^1^Department of Urology, Second Affiliated Hospital, Army Medical University, Chongqing 400037, China; ^2^Wake Forest Institute for Regenerative Medicine, Wake Forest School of Medicine, Winston-Salem, North Carolina 27157, USA

In the article titled “Therapeutic Effects of Human Urine-Derived Stem Cells in a Rat Model of Cisplatin-Induced Acute Kidney Injury In Vivo and In Vitro” [1], the authors identified that the incorrect figure was mistakenly uploaded in Figure 1(d). The authors have provided the corrected Figure 1 as below and confirm that this does not affect the results and conclusion of the article.

## Figures and Tables

**Figure 1:**
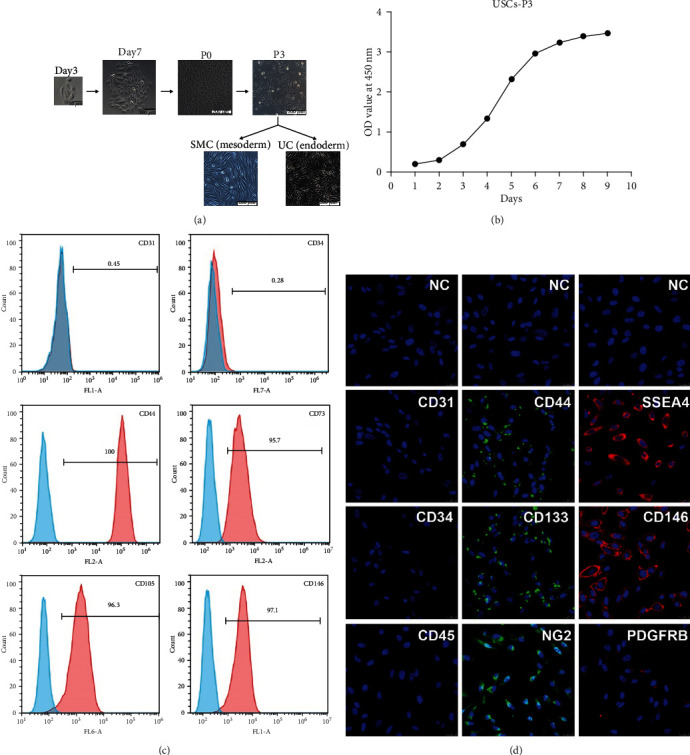
Growth characteristics of USCs. (a) The morphology of USCs by passage and differentiation. Single, small, compact rice grain-like cells were observed on the third day after initial seeding, and they formed a colony on the seventh day. The cells were considered to be at P0 when the confluence reached 70-80% and were passaged to the next generation. The USCs maintained the rice grain-like morphology after several passages, and USCs from the P3 generation were induced to differentiate into SMCs and UCs. The cells showed an elongated and spindle-shaped morphology after SMC differentiation and a cobblestone-shaped morphology after UC differentiation. Scale bar: 50 *μ*m, 100 *μ*m, and 200 *μ*m. (b) The growth curve of USCs from the P3 generation. (c) Detection of surface markers in USCs using flow cytometry. USCs did not express hematopoietic stem cell markers (CD31: 0.45%, CD34: 0.28%) but expressed MSC markers (CD44: 100%, CD73: 97.1%, and CD105: 96.3%) and pericyte markers (CD146: 95.7%). (d) Detection of surface markers in USCs using IF. USCs did not express hematopoietic stem cell markers (CD31, CD34, and CD45) but did express MSC markers (CD44 and CD133), the ESC marker SSEA4, and pericyte markers (CD146, PDGFRB, and NG2). NC: negative control; PDGFRB: platelet-derived growth factor beta-receptor; NG2: neural/glial antigen 2. Scale bar: 25 *μ*m.
